# A Gain-of-Function Cleavage of TonEBP by Coronavirus NSP5 to Suppress IFN-β Expression

**DOI:** 10.3390/cells13191614

**Published:** 2024-09-26

**Authors:** Hyun Park, Sang Min Lee, Su Ji Jeong, Yeong Cheon Kweon, Go Woon Shin, Whi Young Kim, Whaseon Lee-Kwon, Chan Young Park, Hyug Moo Kwon

**Affiliations:** 1 Department of Biological Sciences, Ulsan National Institute of Science and Technology, Ulsan 44919, Republic of Korea; skyline@unist.ac.kr (H.P.); smlee95@unist.ac.kr (S.M.L.); tnwl0623@unist.ac.kr (S.J.J.); kyc4658@unist.ac.kr (Y.C.K.);; 2 Biomedical Engineering, Ulsan National Institute of Science and Technology, Ulsan 44919, Republic of Korea

**Keywords:** SARS-CoV-2, innate immunity, *IFN-β* promoter, gain of function

## Abstract

Human coronaviruses (HCoVs) modify host proteins to evade the antiviral defense and sustain viral expansion. Here, we report tonicity-responsive enhancer (TonE) binding protein (TonEBP) as a cellular target of HCoVs. TonEBP was cleaved into N-terminal and C-terminal fragments (TonEBP NT and TonEBP CT, respectively) by NSP5 from all the HCoVs tested. This cleavage resulted in the loss of TonEBP’s ability to stimulate the TonE-driven transcription. On the other hand, TonEBP NT promoted viral expansion in association with the suppression of *IFN-β* expression. TonEBP NT competed away NF-κB binding to the PRD II domain on the *IFN-β* promoter. A TonEBP mutant resistant to the cleavage by NSP5 did not promote the viral expansion nor suppress the *IFN-β* expression. These results demonstrate that HCoVs use a common strategy of targeting TonEBP to suppress the host immune defense.

## 1. Introduction

Coronaviruses are enveloped viruses with a single-stranded, positive-sense RNA genome of 26–32 kb in size [1]. The genome encodes structural proteins including the spike (S), envelope (E), membrane (M), and nucleocapsid (N); accessory proteins 3, 6, 7a, 7b, 8, 9b, 10b, 13, and 14; and the large polyprotein 1ab (pp1ab). pp1ab is further processed into 16 individual non-structural proteins (NSPs) by papain-like proteases (NSP3) and 3C-like proteases (NSP5) for viral replication and transcription.

Coronaviruses infect primarily birds and mammals including humans. HCoVs such as HCoV-229E, HCoV-NL63, HCoV-OC43, and HCoV-HKU1 cause mild common cold [2]. Since 2002, three new HCoVs causing severe disease have emerged—severe acute respiratory syndrome (SARS), Middle East respiratory syndrome (MERS), and SARS-CoV-2 [3]. While outbreaks of SARS and MERS were relatively limited, SARS-CoV-2 caused a global pandemic for more than 3 years with more than 6.5 million deaths [4]. Although the SARS-CoV-2 pandemic ended, SARS-CoV-2 evolution is still very much in progress as new variants continue to emerge and replace old ones [5]. Given the extraordinarily efficient evolution of SARS-CoV-2, it is likely that new species of HCoV will emerge in the future.

Upon SARS-CoV-2 infection of nasal, bronchial, and lung epithelial cells, the innate immune response is initiated by the sensing of replication-intermediate double-stranded RNA by retinoic acid-inducible gene 1 (RIG-I) and anti-melanoma differentiation-associated gene 5 (MDA5) [6,7]. Due to proteolytic inactivation of RIG-I after the infection (see below), MDA5 activates the RIG-I-like receptor (RLR) signaling cascade, inducing the activation of two central transcription factors, interferon (IFN) regulatory factor 3 (IRF3) and NF-κB, leading to the transcriptional induction of IFN-β [7]. IFN-β functions in both autocrine and paracrine manners to upregulate hundreds of IFN-stimulated genes that exert antiviral effects, either directly or indirectly through diverse mechanisms [8,9]. SARS-CoV-2 uses a variety of strategies to evade the IFN-mediated immune response and establish a productive infection by hijacking host proteins [6]. One of the main strategies used for host immune evasion is the cleavage, and inactivation, of host proteins by SARS-CoV-2 NSP5. SARS-CoV-2 NSP5 cleaves RIG-I, eukaryotic translation initiation factor 4G (eIF4G), and NF-κB essential modulator (NEMO) to repress IFN-β production [10,11,12]. In addition, SARS-CoV-2 NSP5 impairs inflammasome signaling by cleaving NOD-like receptor protein 1 (NLRP1) and NLRP12 [13,14].

TonEBP, also known as NFAT5 (nuclear factor of activated T cell 5), is a transcription factor of the Rel family with homology to NF-κB [15]. TonEBP was initially discovered as a central regulator of cellular responses to hypertonic stress, a role it plays by binding to TonEs. Recent studies have revealed that TonEBP is a versatile stress protein associated with a viral infection. TonEBP is a proteolytic target of coxsackievirus B3 (CVB3) protease 2A [16]. This cleavage promotes CVB3 replication and tissue damage due to the reduced expression of inducible nitric oxide synthase in cardiac myocytes. Thus, TonEBP is an antiviral protein against CVB3 infection in the heart, but it is disabled after CVB3 infection. On the other hand, TonEBP is a proviral protein for lymphocytic choriomeningitis virus replication [17]. It engages with the IFN-β promoter in a Toll-like receptor 3-dependent manner, thereby hindering IRF3 binding and suppressing IFN-β promoter activity, ultimately dampening innate immune responses. Here, we find that NSP5 from several HCoVs cleaves TonEBP. One of the cleavage products, TonEBP NT directly binds to the IFN-β promoter and suppresses *IFN-β* gene expression.

## 2. Materials and Methods

### 2.1. Cell Culture and Transfection

A549 (CCL-185), HEK293T (CRL-1573), U2OS (HTB-96), and Vero-E6 (CRL-1586) cells were obtained from the American Type Culture Collection (Manassas, VA, USA). HEK293T, U2OS, and Vero-E6 cells were cultured in Dulbecco’s modified Eagle’s medium supplemented with 10% fetal bovine serum (FBS), whereas A549 cells were cultured in Roswell Park Memorial Institute-1640 medium with 25 mM HEPES and 10% FBS, at 37 °C, in an incubator with 5% CO_2_. For transient transfection, HEK293T cells were transfected at 70% confluence using jetPRIME^®^ (Polyplus-transfection, Illkirch-Graffenstaden, France) according to the manufacturer’s instructions.

### 2.2. Plasmids

The TonE-driven Photinus luciferase plasmid has been described previously [15]. The Renilla luciferase plasmid (pRL-TK) was purchased from Promega (Madison, WI, USA). Plasmids expressing individual SARS-CoV-2 NSPs were cloned by gateway cloning, using the corresponding donor vector (#141255–#141269, Addgene, Watertown, MA, USA) and a CMV-promoter-driven expression vector. Transient expression plasmids of NSP5s from the 229E, NL63, OC43, HKU9, and SARS-CoV-2 strains have been reported previously [18]. To compare SARS-CoV-2 NSP5 wild type and C145A mutant, EF-1 alpha-promoter-driven expression vector (#141370, #141371) were purchased from Addgene.

The plasmid encoding His-SARS-CoV-2 NSP5 was obtained from Addgene (m4133256). For substitution mutations in TonEBP, stie-directed mutagenesis was carried out using KOD Plus (KOD-201, TOYOBO, Osaka, Japan), according to the manufacturer’s instructions. The plasmid encoding the N-terminal fragment of TonEBP generated by SARS-CoV-2 NSP5 cleavage was cloned into the pCMV-tag 2B vector (#211172, Agilent Technologies, Santa Clara, CA, USA) by amplification and standard restriction cloning.

The *IFN-β* promoter luciferase reporter (#102597), as well as transient expression vectors for MDA5 (#52876), MAVS (#52135), TBK1 (#131792), IRF3 (#127663), and p65 (#111192), were obtained from Addgene. For generation of the expression vector for IRF3-5D, site-directed mutagenesis was performed using KOD Plus, according to the manufacturer’s instructions.

### 2.3. Viral Infection

The human coronavirus HCoV-OC43 (betacoronavirus 1, KBPV-VR-8) was obtained from Korea Bank for Pathogenic Viruses (Seoul, Republic of Korea) and stored at −80 °C. Viral infection titers were measured using a focus-forming assay on Vero-E6 cells. Vero-E6 cells were grown to confluence in 96-well plates. Cells at 90% confluence were infected with 10-fold serial dilutions of a HCoV-OC43 stock solution for 3 h at 33 °C in an incubator maintained at 5% CO_2_ without serum and with gentle rocking. After infection, the cells were cultured for 24 h in regular medium supplemented with 2% FBS. The cells were then fixed with 4% paraformaldehyde and permeabilized with 0.5% Triton™ X-100. After blocking with 3% bovine serum albumin, immunofluorescence was performed using a mouse monoclonal antibody against HCoV-OC43 N protein (MAB9012, Merck Millipore, Burlington, MA, USA). Antibody labeling was detected using a secondary antibody conjugated with Alexa Fluor™ 633 (Invitrogen, Carlsbad, CA, USA). Foci were visualized and counted using a fluorescence microscope (IN Cell Analyzer 2500, Cytiva, Marlborough, MA, USA).

A549, HEK293T, and U2OS cells were incubated with HCoV-OC43 for 3 h at different multiplicities of infection without serum. After removing the infectious solution, the cells were washed with serum-free medium and maintained in regular medium containing 2% FBS. The infected cells were subjected to Western blot, luciferase assays, or immunofluorescence.

### 2.4. Immunoblot Analysis and Immunoprecipitation

For immunoblot analysis, the cells were washed three times with cold PBS and then lysed with an appropriate volume of 0.1% sodium dodecyl sulfate (SDS) lysis buffer (50 mM Tris-HCl pH 7.5, 150 mM NaCl, and 1% Triton™ X-100). After rotation at 4 °C for 10 min, the lysates were centrifuged at 13,000 rpm and 4 °C for 10 min, following which the supernatant was collected. SDS dye (4×; 0.2 M Tris-HCl pH 6.8, 8% SDS, 40% glycerol, and 0.04% bromophenol blue) was then added to the supernatant and incubated at 95 °C for 10 min.

For immunoprecipitation, the cells were washed three times with cold PBS and then lysed with an appropriate volume of lysis buffer (25 mM Tris-HCl pH 7.4, 150 mM NaCl, and 1% NP-40). After rotation at 4 °C for 10 min, the lysates were centrifuged at 13,000 rpm and 4 °C for 10 min and the supernatant was incubated at 4 °C with anti-Flag M2 agarose beads (A2220, Merck Millipore) overnight. SDS dye (4×; 0.2 M Tris-HCl pH 6.8, 8% SDS, 40% glycerol, and 0.04% bromophenol blue) was then added to the lysates and the immunoprecipitated samples were incubated at 95 °C for 5 min.

Samples were separated using 10% sodium dodecyl sulfate–polyacrylamide gel electrophoresis and transferred onto polyvinylidene difluoride or nitrocellulose membranes. The membranes were blocked with Tris-buffered saline + 0.1% Tween 20 supplemented with 7% skim milk for 1 h. After blocking, the membranes were incubated overnight with the following primary antibodies: rabbit anti-TonEBP (1:3000 in blocking solution) [15]; monoclonal mouse anti-HCoV-OC43 N protein (1:1000 in blocking solution; MAB9012) from Merck Millipore; monoclonal mouse anti-β-actin (1:1000 in blocking solution; SC-47778) from Santa Cruz Biotechnology (Santa Cruz, CA, USA); polyclonal rabbit anti-GAPDH (1:10,000 in blocking solution; 10494-1-AP), monoclonal mouse anti-GFP (1:10,000 in blocking solution; 66002-1-Ig), and monoclonal mouse anti-His (1:10,000 in blocking solution; 66005-1-Ig) from ProteinTech (Rosemont, IL, USA); monoclonal mouse anti-Flag (1:1000 in blocking solution; F1804) from Sigma-Aldrich (St. Louis, MO, USA); and monoclonal rabbit anti-TBK1/NAK (1:1000 in blocking solution; #3504), anti-phospho-TBK1/NAK (1:1000 in blocking solution; #5483), anti-IRF3 (1:1000 in blocking solution; #4302), and anti-phospho-IRF3 (1:1000 in blocking solution; #29047) from Cell Signaling Technology (Danvers, MA, USA). After several washes with Tris-buffered saline + 0.1% Tween 20, each blot was further incubated with the appropriate secondary antibody, either goat anti-mouse (1:10,000 in blocking solution; AP124P) or anti-rabbit (1:10,000 in blocking solution; AP307P) IgG conjugated to horseradish peroxidase (both from Millipore). The polyvinylidene difluoride blots were detected via chemiluminescence using Clarity™ Western ECL substrate solution (Bio-Rad, Hercules, CA, USA), whereas nitrocellulose blots were incubated with fluorescence-conjugated secondary antibodies: IRDye^®^ 800CW goat anti-rabbit (1:10,000 in blocking solution; 926-32211) or 680RD goat anti-mouse (1:10,000 in blocking solution; 926-68070) (LI-COR, Lincoln, NE, USA). Fluorescent blot detection was performed using an Odyssey^®^ CLx Imaging System (LI-COR).

### 2.5. Luciferase Assay

HEK293T cells were co-transfected with the indicated luciferase constructs of TonE-driven Photinus [15], or *IFN-β* promoter luciferase reporter (#102597, Addgene) and the Renilla luciferase plasmid (pRL-TK), which was used as an internal control for transfection efficiency. For the *IFN-β* promoter luciferase assay, control plasmid or plasmids expressing MDA5, MAVS, TBK1, IRF3-5D, or p65 were used. Luciferase assays were performed using the Dual-Luciferase^®^ Reporter Assay System (Promega). For each condition, the luciferase activity was measured using samples taken from triplicate wells using a 96-well automated luminometer (Bio-Rad). Results were calculated as the ratio of firefly luciferase activity to Renilla luciferase activity.

### 2.6. Sequence Alignment

NSP5 protein sequences of coronaviruses were derived from available ORF1ab sequences: 229E (NP_073549.1), NL63 (YP_003766.2), OC43 (YP_009555238.1), HKU9 (YP_001039970.1), and SARS-CoV-2 (YP_009724389.1). These NSP5 protein sequences were aligned and analyzed using SnapGene version 7.2.1 (GSL Biotech LLC, San Diego, CA, USA).

### 2.7. Immunofluorescence Staining

HEK293T cells were seeded onto a 4-well chamber (#177429, Thermo Scientific, Waltham, MA, USA) and transfected with the indicated plasmids. The transfected cells were incubated at 37 °C for 48 h and then fixed with 4% paraformaldehyde, permeabilized with 0.5% Triton™ X-100, and blocked with 3% bovine serum albumin for 1 h. The nuclei were stained with DAPI (4′,6-diamidino-2-phenylindole). The cells were observed under a Zeiss LSM 780 confocal microscope (Carl Zeiss, Jena, Germany).

### 2.8. U2OS Stable Cell Line Construction

HEK293T cells were seeded into 6-well plates at a density of 2 × 10^5^ cells/mL and then transfected with lentiviral constructs (YFP and YFP-TonEBP NT) and packaging plasmids (VSVg, p8.2). After transfection for 16 h, the cells were washed with PBS and fresh medium was added. After 48 h of transfection, the lentiviral supernatants were collected and used to treat U2OS cells. After 48 h of lentiviral infection, U2OS cells were subjected to treatment with 2 μg/mL puromycin to select single-cell colonies for further experiments. Lentiviral constructs were cloned into the pLIX403-EYFP-ccdB-Puro plasmid (#158549; Addgene).

### 2.9. RNA Extraction and Quantitative Real-Time PCR

Total cellular RNA was extracted by phenol–chloroform extraction using RiboEX™ (GeneAll, Seoul, Republic of Korea), according to the manufacturer’s instructions. A total of 1 µg of RNA was converted into cDNA by reverse transcription using the ReverTra Ace-α^®^ cDNA Synthesis Kit (TOYOBO). mRNA expression levels were detected by quantitative real-time PCR using SYBR^®^ Green PCR Master Mix (TOYOBO). Actin mRNA was used as an endogenous control. All experiments were performed in triplicate. The primers used are given below:

Human *IFN-β* forward, AAACTCATGAGCAGTCTGCA;

Human *IFN-β* reverse, AGGAGATCTTCAGTTTCGGAGG;

Human *GAPDH* forward, CTGAACGGGAAGCTCACTGGCATG;

Human *GAPDH* reverse, AGGTCCACCACCCTGTTGCTGTAGC;

HCoV-OC43 *Nucleocapsid* forward, CGATGAGGCTATTCCGACTAGGT;

HCoV-OC43 *Nucleocapsid* reverse, CCTTCCTGAGCCTTCAATATAGTAACC.

### 2.10. Extracellular OC43 Titer Assay

To define the standard curve for the extracellular HCoV-OC43 titer, we extracted viral RNA from 150 µL of 10-fold serial dilutions of the HCoV-OC43 stock using the PURY Viral RNA Mini Kit (GenDEPOT, Katy, TX, USA). The cDNAs of viral RNA were synthesized by reverse transcription using the ReverTra Ace-α^®^ cDNA Synthesis Kit, with random primers. The synthesized cDNAs was analyzed using quantitative real-time PCR, and the Cq values for each titer were used to define the standard curve. For the viral titer assay, 150 µL of extracellular medium containing HCoV-OC43 was collected at 68 h post-infection. Viral RNA was isolated, converted into cDNA, and analyzed using quantitative real-time PCR. Cq values were used to calculate the extracellular viral titers using a standard curve.

### 2.11. Focus-Forming Assay

Cells were seeded at a density of 2 × 10^5^ cells/mL and then treated with doxycycline the following day. After 24 h of induction of protein expression, the cells were infected with the OC43 virus at a multiplicity of infection of 0.001. After 2 h of infection, the cells were washed more than three times with PBS, following which 2% FBS containing 1% low-melting agarose was added to cover the surface. A 10% paraformaldehyde solution was added above the agarose layer and cells were fixed for more than 1 h at 24 h post-infection. After fixation, the agarose layer was removed and the cells were permeabilized with 0.1% Triton™ X-100 for 10 min. Cells were blocked with 3% bovine serum albumin for 2 h and then incubated overnight with a primary antibody against OC43 N protein (1:1000 in blocking solution; MAB9012). After several washes with PBS, the cells were incubated with a fluorescence-conjugated secondary antibody (1:1000 in blocking solution; anti-mouse Alexa Fluor™ 594; A-11032; Thermo Fisher Scientific, Waltham, MA, USA) for 2 h. Nuclear staining was performed using Hoechst solution for 10 min. The number of OC43-infected cells per foci was analyzed using an IX83 inverted microscope (Olympus, Tokyo, Japan).

### 2.12. Enzyme-Linked Immunosorbent Assay

The IFN-β concentrations in HEK293T cell culture supernatants were determined using a Human IFN-β Assay Kit (DIFNB0, R&D Systems, Minneapolis, MN, USA), according to the manufacturer’s instructions. The absorbance was recorded at a wavelength of 450 nm using a Tecan infinite™ M200 plate reader (Tecan, Männedorf, Switzerland), with wavelength correction by subtracting the absorbance at 540 nm.

### 2.13. Chromatin Immunoprecipitation (ChIP) Analysis

For ChIP analysis, cells were crosslinked with 1% formaldehyde (F8775; Sigma-Aldrich) for 10 min and quenched with 0.125 M glycine for 5 min, at room temperature. ChIP was performed using a ChIP Assay Kit (17-295; Sigma-Aldrich), according to the manufacturer’s instructions. Antibodies against TonEBP [15], and normal rabbit IgG (2729; Cell Signaling Technology) were used. Input and immunoprecipitated DNA were purified using a QIAquick^®^ PCR Purification Kit (Qiagen, Hilden, Germany). The purified input and immunoprecipitated DNA were detected by real-time PCR, using specific primers. The immunoprecipitated DNA from each sample was calculated as a percentage of the respective chromatin input and normalized to the control experiment for each condition. The following primers were used:

*IFN-β* promoter forward, AGGACCATCTCATATAAATAGGCCATACCC;

*IFN-β* promoter reverse, ACTGAAAATTGCTGCTTCTTTGTAGGAATC.

### 2.14. Protein Expression and Purification

Recombinant TonEBP sNT or p65 was expressed in the N-terminal His-fused form in *E. coli* BL21 cells (DE3). BL21 transformed with the expression plasmid of TonEBP sNT or p65 were grown in 2.0 L Luria Broth supplemented with the appropriate antibiotics, at 37 °C, with shaking, until the optical density at the wavelength of 600 nm reached a value of ~0.6. The BL21 culture was then cooled to 18 °C, and protein expression was induced by addition of 1.0 mM isopropyl β-D-1-thiogalactopyranoside. The cells were harvested after induction for 20 h by centrifugation at 4000× *g*, 4 °C, for 20 min.

The cells were resuspended in pre-cooled buffer containing 20 mM Tris-HCl pH 8.0, 150 mM NaCl, and 1 mM dithiothreitol (DTT). The resuspended cells were then lysed by sonication for 9 cycles (20 s on and 30 s off) at an amplitude of 35, on ice, following which the cell debris were removed by centrifugation at 17,000× *g* and 4 °C for 1 h. The resulting supernatant was supplemented with 1 mL pre-equilibrated HisPur™ Ni-NTA resin (Thermo Scientific) and 20 mM imidazole and incubated overnight at 4 °C. Following that, the samples were loaded onto a gravity column and washed three times with 10 mL of wash buffer containing 20 mM Tris-HCl pH 8.0, 150 mM NaCl, 20 mM imidazole, and 1 mM DTT. The protein was then eluted in 1 mL of elution buffer containing 20 mM Tris-HCl pH 8.0, 150 mM NaCl, 200 mM imidazole, and 1 mM DTT. The eluted proteins were loaded onto a Superdex™ 75 Increase 10/300 GL (29-1487-21; Cytiva) gel filtration column equilibrated with PBS pH 7.4. The protein samples were finally aliquoted and stored at –80 °C.

### 2.15. Electrophoretic Mobility Shift Assay (EMSA)

The indicated amounts of purified His-TonEBP sNT or -p65 and 100 nM of indicated Cy5.5-conjugated probe were incubated at room temperature in the dark for 25 min, in a total volume of 20 μL in binding buffer (10 mM Tris-HCl pH 7.5, 10 mM NaCl, 40 mM KCl, 1 mM MgCl_2_, 1 mM EDTA, 1 mM DTT, and 50 μg/mL bovine serum albumin). In competition assays, p65 was incubated with probe for 20 min prior to incubation with TonEBP sNT for 25 min. After incubation, the resulting mixtures were subjected to electrophoresis on a 0.8% agarose gel in 0.5× Tris-borate-EDTA (45 mM Tris, 45 mM borate, 1 mM EDTA pH 8.3). The gel was exposed and imaged using the Odyssey^®^ CLx Imaging System (Li-COR). The following probes were used:

Probe IRF3/p65, Cy5.5-ATAGGAAAACTGAAAGGGAGAAGTGAAAGTGGGAAATTCC;

Probe IRF3, Cy5.5-GAAAACTGAAAGGGAGAAGTGAAAGTG;

Probe p65, Cy5.5-AATGTAAGTGGGAAATTCCTCTGAATA.

### 2.16. Surface Plasmon Resonance Assay

The affinities between TonEBP sNT or p65 and *IFN-β* promoter were measured at room temperature using a Biacore™ T200 system (Cytiva) with a streptavidin-coated sensor chip, Series S Sensor Chip SA (BR100531, Cytiva). The biotin-tagged *IFN-β* promoter probe was immobilized on the chip with a concentration of 10 nM and at a flow rate of 10 μL/min. A blank channel was left untreated. The same procedure was used to immobilize biotin as a negative control. For all measurements, the same running buffer, consisting of 10 mM HEPES, 150 mM NaCl, 3 mM EDTA, and 0.05% Surfactant P20, was used. Serially diluted recombinant TonEBP sNT or p65 was then allowed to flow through the chip surface, at a flow rate of 50 μL/min. Data were analyzed using the T200 BIA evaluation software (GE Healthcare, Arlington Heights, IL, USA), with curve fitting to the data obtained using the 1:1 binding model. The following probes were used:

Probe IRF3/p65, Biotin-ATAGGAAAACTGAAAGGGAGAAGTGAAAGTGGGAAATTCC;

Probe IRF3, Biotin-GAAAACTGAAAGGGAGAAGTGAAAGTG;

Probe p65, Biotin-AATGTAAGTGGGAAATTCCTCTGAATA.

### 2.17. Statistics

Statistical analyses were performed using Prism version 9 (GraphPad Software, San Diego, CA, USA).

## 3. Results

### 3.1. Human Coronavirus NSP5s Cleave TonEBP and Inhibit Its Transcriptional Activity

**Figure 1:**
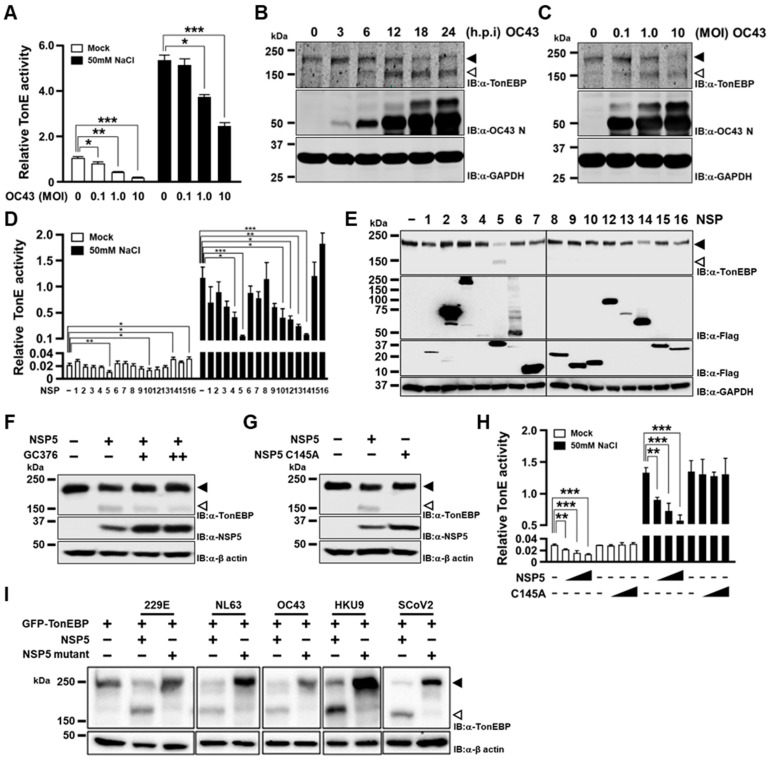
TonEBP is cleaved by human coronavirus NSP5. (**A**) HEK293T cells were transfected with a TonE-driven Photinus luciferase plasmid. pRL-TK plasmid driving the expression of Renilla luciferase was co-transfected to normalize transfection efficiency. After 16 h, the cells were infected with HCoV-OC43 for 24 h at different MOIs indicated. The cells were then treated without (mock) or with 50 mM NaCl for 6 h before analyses of Photinus and Renilla luciferase activity to calculate TonE activity. (**B**) A549 cells were infected with HCoV-OC43 at an MOI of 20 for up to 24 h, as indicated. The cells were then analyzed by immunoblotting (IB) for TonEBP, OC43 N, and GAPDH. Full-length TonEBP and cleaved TonEBP are marked by solid and open arrowhead, respectively. (**C**) A549 cells were infected with HCoV-OC43 for 24 h at MOI up to 10, as indicated, before immunoblotting. (**D**) HEK293T cells were transfected for 16 h with the TonE-driven luciferase plasmid and pRL-TK, along with an empty vector (marked “−“) or a plasmid expressing one of NSPs (NSP1 to NSP16 except NSP11, as marked 1 to 16 except 11) from SARS-CoV-2. The cells were then treated without or with 50 mM NaCl and luciferase was measured. (**E**) Cells in isotonic conditions (mock) were transfected with one of the SARS-CoV-2 NSPs followed by immunoblotting for TonEBP, Flag, and GAPDH. (**F**) Cells were transfected with the plasmid expressing SARS-CoV-2 NSP5 for 4 h and treated with 0, 30, or 50 μM of GC376 for an additional 20 h. (**G**) Cells were transfected with a plasmid expressing either wild-type or catalytically inactive mutant C145A SARS-CoV-2 NSP5. (**H**) Cells were transfected with the TonE-driven luciferase plasmid and pRL-TK, along with the empty vector (-) or increasing amounts of the plasmid expressing SARS-CoV-2 NSP5 or C145A for 16 h. The transfected cells were treated without or with 50 mM NaCl for 6 h before analyses of luciferases. (**I**) Cells were co-transfected with plasmids expressing GFP-TonEBP (GFP is conjugated to the N-terminus of TonEBP) and either wild type or catalytically inactive mutant of NSP5 from HCoV-229E, HCoV-NL63, HCoV-OC43, bat CoV-HKU9 or SARS-CoV-2 (SCoV2). Full-length GFP-TonEBP and cleaved GFP-TonEBP are marked by filled and open arrowhead, as in (**E**–**G**) above. Data are shown as the mean ± SEM, *n* = 3; * *p* < 0.033, ** *p* < 0.002, and *** *p* < 0.001 using *t*-tests (**A**,**D**,**H**). MOI, multiplicity of infection; GFP, green fluorescent protein; NSP, non-structural protein.

Given the diverse effects of TonEBP on viral infection, we first asked whether TonEBP was affected by coronaviruses. When HEK293T cells were infected by HCoV-OC43 with increasing multiplicity of infection (MOI), we found that the TonE-driven transcription was inhibited in a dose-dependent manner (Figure 1A). As expected, the TonE-driven transcription was stimulated by hypertonicity made by adding 50 mM NaCl, which was also inhibited by HCoV-OC43 infection. We examined TonEBP in a lung epithelial cell line A549 as well as in HEK293T cells using the antibody raised against the N-terminus of TonEBP [15]. Infection by HCoV-OC43 was associated with the time- (Figure 1B) and dose-dependent (Figure 1C) disappearance of TonEBP. The disappearance of TonEBP was accompanied by the appearance of a smaller TonEBP, which might represent a cleaved TonEBP (see below).

We next asked whether the inactivation of TonEBP was mediated by any of the NSPs. We individually transfected SARS-CoV-2 NSP1 to 16 except 11 and found that NSP5 and NSP10 inhibited the TonE-driven transcription under isotonic conditions (Figure 1D). On the other hand, under hypertonic conditions NSP5 and NSP14 exhibited strongest inhibition of the transcription. Immunoblotting revealed that TonEBP expression was prominently reduced by both NSP5 and NSP14 (Figure 1E). We decided to focus on NSP5 because it showed consistent inhibitory effects and it appeared to cleave TonEBP producing a smaller protein as in Figure 1B,C.

The cleavage of TonEBP by NSP5 was sensitive to GC376, an inhibitor of NSP5 (Figure 1F), and a catalytically inactive mutant C145A did not cleave TonEBP (Figure 1G), demonstrating that the proteolytic activity of NSP5 was responsible. The TonE-driven transcription was suppressed by NSP5 in a dose-dependent manner but not suppressed by NSP5 C145A both under isotonic and hypertonic conditions (Figure 1H), as expected. Finally, we examined NSP5 from three other HCoVs—HCoV-229E, HCoV-NL63, HCoV-OC43, and a bat CoV-HKU9. Analyses of their sequences revealed that SARS-CoV-2 NSP5 shares 40.8%, 44.3%, 48.0% and 52.0% amino acid sequence identity with 229E, NL63, OC43 and HKU9, respectively (Figure S1). The catalytic dyad residues His41 and Cys145 of SARS-CoV-2 NSP5 are essential for protease activity and conserved among these five coronaviruses [19]. All of them cleaved TonEBP in a manner dependent on the critical C residue in their catalytic site (Figure 1I) like NSP5 from SARS-CoV-2. Taken together, these data show that NSP5s from HCoVs cleave TonEBP and inhibit its transcriptional activity.

### 3.2. SARS-CoV-2 NSP5 Targets Q1127 within the Transcriptional Activation Domain (TAD) of TonEBP

**Figure 2:**
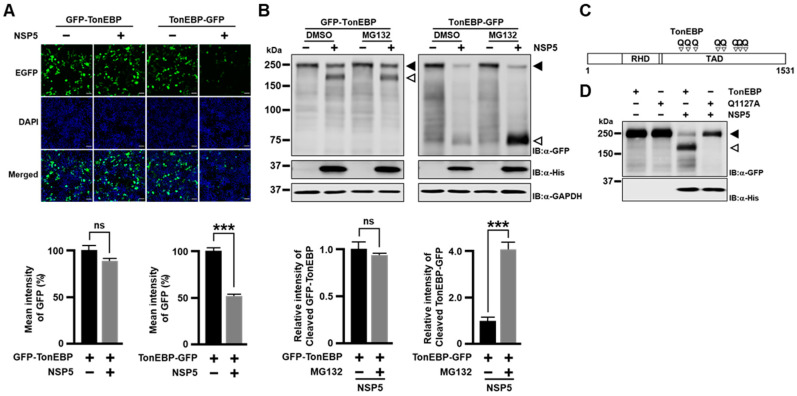
SARS-CoV-2 NSP5 targets Q1127 of TonEBP. (**A**) HEK293T cells were co-transfected with the plasmid expressing either GFP-TonEBP (see above) or TonEBP-GFP (GFP is conjugated to the C-terminus of TonEBP) along with the empty vector or His-tagged SARS-CoV-2 NSP5-expressing vector, as indicated. At 48 h later, the cells were fixed and stained with DAPI. Fluorescence images for GFP, DAPI, and the two merged are shown. Scale bar: 100 µm. GFP fluorescence intensity was quantified as the mean ± SEM, *n* = 3 (bottom). (**B**) Cells were transfected for 24 h with constructs as in (**A**). The cells were then treated for 6 h with vehicle (DMSO) or 10 μM MG132 before immunoblotting. Full-length GFP-TonEBP and cleaved GFP-TonEBP are marked by solid and open arrowhead, respectively. Relative intensity of the cleaved GFP-TonEBP (open arrowhead) was quantified as the mean ± SEM, *n* = 3 (bottom). (**C**) Schematic of TonEBP primary sequence with putative cleavage sites marked with Qs predicted using NetCorona1.0 (with a score higher than 0.35). RHD, Rel-homology domain; TAD, transcription activation domain. (**D**) Cells were transfected with various combinations of plasmids expressing GFP-TonEBP (TonEBP), GFP-TonEBP with Q1127A mutation (Q1127A), and NSP5, as indicated, before immunoblotting. ns *p* > 0.05, and *** *p* < 0.001 using *t*-tests (**A**,**B**).

To visualize both the N-terminal and C-terminal fragments of TonEBP separately, we used two GFP conjugated constructs—GFP-TonEBP and TonEBP-GFP where GFP was conjugated to the N- and C-terminus, respectively. When GFP-TonEBP was expressed, the GFP fluorescence was not affected by co-expression of NSP5 (Figure 2A). On the other hand, the GFP fluorescence was halved when TonEBP-GFP was co-expressed with NSP5. We wondered whether the C-terminal fragment was degraded by proteasome because cleaved protein by SARS-CoV-2 NSP5 such as NLRP1 was known to be degraded by proteasome [13]. To test this, we immunoblotted the transfected cells after treatment with MG132, a proteasome inhibitor (Figure 2B). While the GFP fluorescence of the cleaved fragment from GFP-TonEBP was not affected by MG132, corresponding GFP fluorescence from TonEBP-GFP increased 4 fold, demonstrating that the C-terminal cleavage fragment was degraded by proteasome.

To determine the cleavage site(s) of TonEBP by NSP5, we used the NetCorona web server (https://services.healthtech.dtu.dk/services/NetCorona-1.0/, accessed on 20 December 2022) to predict potential cleavage sites. We identified eight Q residues as putative target sites with scores higher than the threshold of 0.35 (Figure 2C and Figure S2A). All of the Q residues are in the transactivation domain (TAD) [20]. These Q residues were individually mutated to A in appropriate TonEBP fragments (Figure S2B,C). We found that only Q1127 mutant was resistant to cleavage by NSP5 (Figure S2C) suggesting that the Q1127 residue was targeted by NSP5 for cleavage. Next, we performed site-directed mutagenesis to create a Q1127A mutant of GFP-TonEBP. This mutant was resistant to cleavage by NSP5 (Figure 2D), demonstrating that NSP5 targets Q1127 for cleavage. The loss of the TonE-driven transcriptional activity of TonEBP by NSP5 (Figure 1H) can be explained by the inactivation of TAD as Q1127 is in the middle of TAD [20]. The sequence around Q1127 is conserved in other mammals (Figure S2D) suggesting the same cleavage by coronaviruses.

### 3.3. Cleavage of TonEBP Promotes Expansion of HCoV-OC43

**Figure 3:**
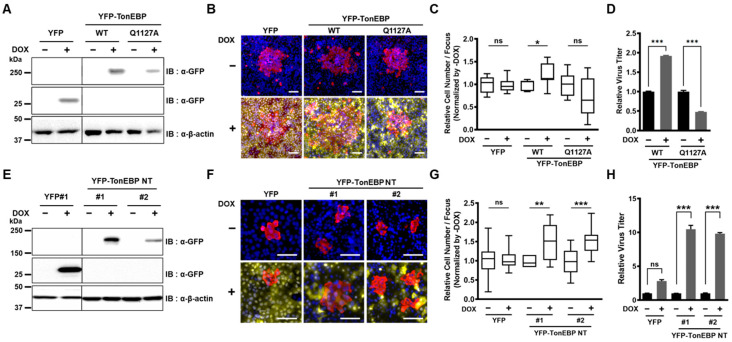
Cleavage of TonEBP promotes expansion of HCoV-OC43. (**A**) U2OS stable cells expressing doxycycline (DOX)-inducible YFP, YFP-TonEBP wild type (WT) or YFP-TonEBP Q1127A were cultured without or with DOX, as indicated, and analyzed by immunoblotting. Anti-GFP antibody was used to detect YFP. (**B**) The cells in (**A**) were infected with HCoV-OC43 at an MOI of 0.001 and covered with 1% low-melting agarose. After 24 h, cell culture plates were fixed and stained with anti-HCoV-OC43 N protein antibody (red) and DAPI (blue). YFP is shown in yellow. Scale bar: 100 µm. (**C**) Number of cells in a single focus of N protein in TonEBP WT and TonEBP Q1127A relative to YFP was shown in box and whisker plots, *n* = 7–11. (**D**) The cells in (**A**) were infected with HCoV-OC43 as in (**B**) and cultured for 68 h. HCoV-OC43 was quantified from the culture media using RT-qPCR. Relative virus titer was normalized to the virus titer of the doxycycline-free condition of each cell line. Mean ± SEM, *n* = 3. (**E**) A single colony of cells stably expressing DOX-inducible YPF (YPF#1) and two colonies expressing U2OS stable cells expressing YFP-TonEBP NT were cultured without or with DOX. (**F**) The cells in (**E**) were cultured and infected with HCoV-OC43 as in (**B**). (**G**) Number of cells in a single focus of N protein in (**E**) was analyzed as in (**C**). Box and whisker plots are shown, *n* = 7–29. (**H**) The cells in (**E**) were infected with HCoV-OC43 as in (**F**) and cultured for 68 h. HCoV-OC43 was quantified from the culture media. Statistical analysis was conducted using one-way ANOVA combined with Bonferroni’s multiple comparisons test; ns *p* > 0.05, * *p* < 0.05, ** *p* < 0.01, and *** *p* < 0.001.

To investigate the role of TonEBP cleavage by NSP5 during coronavirus infection, we generated U2OS cell lines that stably express doxycycline-inducible yellow fluorescent protein (YFP), YFP-TonEBP wild type (WT), or YFP-TonEBP Q1127A (Figure 3A). Using these cell lines, we aimed to examine the effect of TonEBP cleavage on viral replication. We measured the propagation of HCoV-OC43 by utilizing the focus-forming assay (FFA) in the presence or absence of doxycycline. Cells were infected with a multiplicity of infection (MOI) of 0.001 and then covered with low-melting-agarose to restrict viral propagation to cells adjacent to the initially infected cell. Infected cells were labeled with an anti-HCoV-OC43 N protein antibody, and the size of each focus was determined by counting the number of OC43 N-positive cells (Figure 3B). An increase in focus size indicates enhanced viral propagation. Expression of YFP alone did not affect the number of infected cells per focus. However, the number of infected cells per focus significantly increased in cells expressing YFP-TonEBP WT, while a decrease was observed in cells expressing YFP-TonEBP Q1127A (Figure 3C). Additionally, qPCR analysis of the total viral output from infected cells showed that YFP-TonEBP WT expression promoted viral expansion, whereas YFP-TonEBP Q1127A expression significantly reduced it (Figure 3D).

Next, we examined the effects of cleaved TonEBP—the N-terminal fragment of TonEBP (TonEBP NT, i.e., TonEBP 1-1127). We used two independent colonies of U2OS cells expressing doxycycline-inducible TonEBP NT (Figure 3E). The focus-forming assay revealed that the expression of TonEBP NT increased the number of infected cells per focus in both colonies, confirming that cleavage of TonEBP enhances viral propagation (Figure 3F,G). The number of viruses released from infected cells also increased dramatically by the expression of TonEBP NT in both colonies (Figure 3H). Combined with the previous data (Figure 3A–D), these results demonstrate that NSP5-mediated cleavage of TonEBP promotes the expansion of HCoV-OC43.

### 3.4. TonEBP Cleavage Blocks the Antiviral Induction of IFN-β

**Figure 4:**
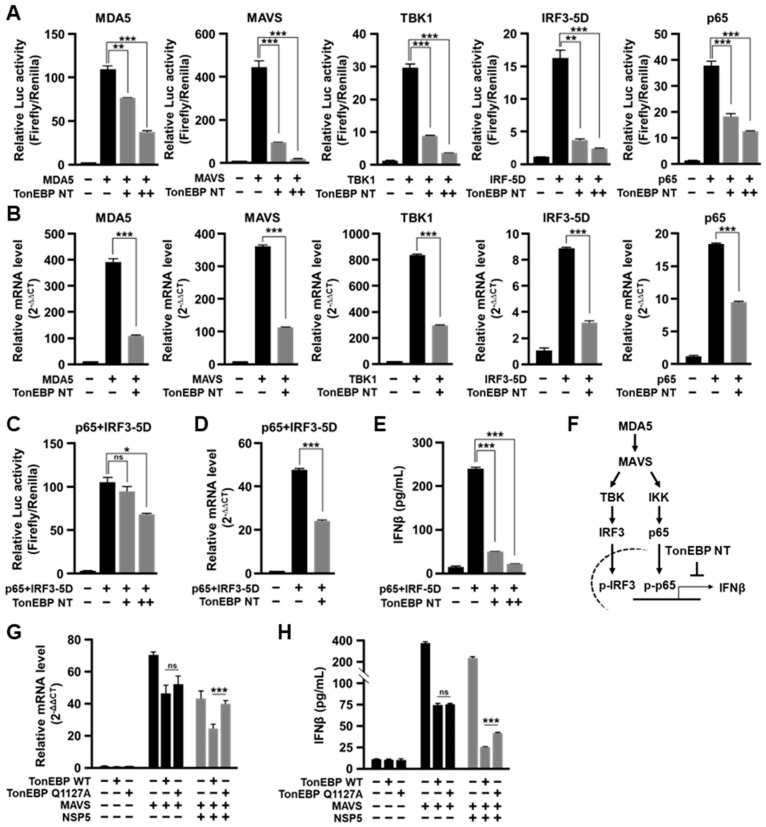
Cleaved TonEBP blocks viral induction of IFN-β. (**A**) HEK293T cells were transfected for 24 h with an *IFN-β* promoter luciferase reporter plasmid and pRL-TK in combination with a plasmid expressing MDA5, MAVS, TBK1, IRF3-5D, or p65 (see (**F**) below for the signaling pathway), along with various amounts of expression plasmid encoding TonEBP NT, as indicated, before analyses of luciferases. (**B**) The cells were transfected for 24 h with one of the plasmids expressing MDA5, MAVS, TBK1, IRF3-5D, or p65, without or with the expression plasmid for TonEBP NT, as indicated. The level of *IFN-β* mRNA was then determined using RT-qPCR. The transcript level of *IFN-β* was normalized to the expression of GAPDH. (**C**) Cells were transfected and analyzed as in (**A**) except that two expression plasmids for IRF3-5D and p65 were used instead of single plasmid for the signaling molecules. (**D**) Cells were transfected and analyzed as in (**B**) except that two expression plasmids for IRF3-5D and p65 were used instead of single plasmid for the signaling molecules. (**E**) Cells were transfected as in (**D**) for 48 h except for variable amounts of the plasmid for TonEBP NT, and IFN-β in the culture media were measured using enzyme-linked immunosorbent assay. (**F**) A model for the inhibitory role of cleaved TonEBP (TonEBP NT) in the MDA5–IFN-β signaling pathway. (**G**) Cells were co-transfected for 24 h with various combinations of four plasmids expressing TonEBP WT, TonEBP Q1127A, MAVS and SARS-CoV-2 NSP5, as indicated. The level of *IFN-β* mRNA was then determined using RT-qPCR. The transcript level of *IFN-β* was normalized to the expression of *GAPDH*. (**H**) Cells were co-transfected for 48 h with various combinations of four plasmids expressing TonEBP WT, TonEBP Q1127A, MAVS and SARS-CoV-2 NSP5, as indicated. IFN-β in the culture media were measured. Data are shown as the mean ± SEM, *n* = 3; ns *p* > 0.05, * *p* < 0.033, ** *p* < 0.002, and *** *p* < 0.001 using *t*-tests.

To understand the dramatic stimulation of HCoV-OC43 expansion by TonEBP NT, we wondered whether the antiviral induction of IFN-β was affected. We decided to examine the MDA5-IFN-β signaling pathway (Figure 4F) because it is activated by SARS-CoV-2 infection in the airway epithelia [6,7]. For this, we first examined the *IFN-β* promoter luciferase reporter. The *IFN-β* promoter was stimulated by expression of each of the molecules in the MDA5-IFN-β signaling pathway as expected—MDA5, MAVS (mitochondrial antiviral signaling), TBK1 (TANK-binding kinase 1), IRF3-5D (a constitutively active form of IRF3), or p65 (NF-κB) (Figure 4A). All of these were inhibited by TonEBP NT in a dose-dependent manner. The changes in the *IFN-β* promoter activity (Figure 4A) were mirrored by corresponding changes in the expression of *IFN-β* mRNA (Figure 4B). When IRF3-5D and p65 was combined, the stimulation of the *IFN-β* promoter and *IFN-β* mRNA was synergistically elevated (Figure 4C,D), which was also suppressed by TonEBP NT. These changes were paralleled by corresponding changes in the production and release of IFN-β (Figure 4E). TonEBP NT appears to suppress sites downstream of activated IRF3 and p65 (Figure 4F) because the inhibitory effects of TonEBP NT was not associated with changes in the expression of signaling molecules of the MDA5-IFN-β pathway nor phosphorylation of IRF3 and TBK1 (Figure S3).

To examine the role of TonEBP cleavage by NSP5 on the MDA5-IFN-β signaling pathway, we compared TonEBP WT vs. TonEBP Q1127A for their ability to suppress *IFN-β* mRNA (Figure 4G) and IFN-β production (Figure 4H). In the absence of NSP5, IFN-β mRNA and protein levels were suppressed by expression of TonEBP WT or TonEBP Q1127A as reported earlier [17]. On the other hand, when NSP5 was co-expressed, *IFN-β* mRNA and protein levels were reduced by TonEBP WT compared to TonEBP Q1127A. These data demonstrate that the TonEBP cleavage by NSP5 blocks the antiviral induction of IFN-β, leading to enhanced expansion of coronavirus infection.

### 3.5. TonEBP NT Suppresses IFN-β Gene Expression by Competing Away p65 at the PRD II Domain on the IFN-β Promoter

**Figure 5:**
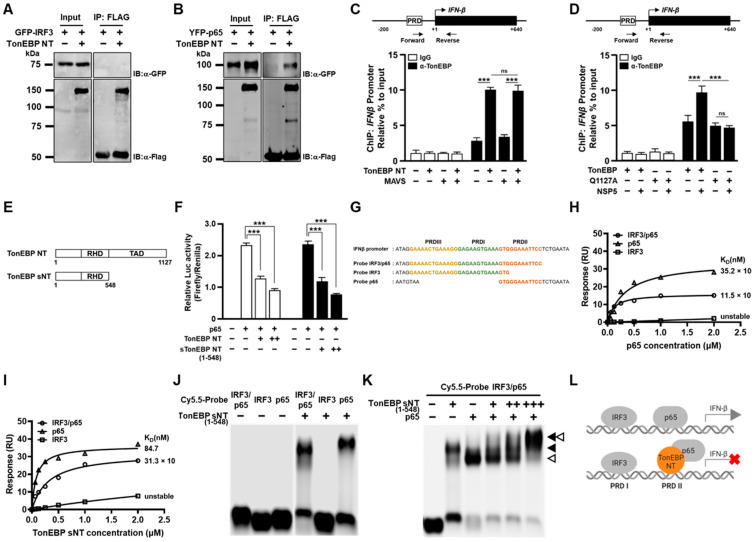
Cleavage of TonEBP promotes binding to the PRD II domain of the *IFN-β* promoter. (**A**) HEK293T cells were co-transfected for 16 h with a plasmid expressing GFP-IRF3 without or with the plasmid expressing Flag-TonEBP NT. Cell lysates (Input) were immunoprecipitated (IP) with anti-Flag antibody. (**B**) Cells were transfected and analyzed as in (**A**) except that YPF-p65 was expressed instead of GFP-IRF3. (**C**) HEK293T cells were co-transfected for 24 h with various combinations of two plasmids expressing Flag-TonEBP NT and MAVS, as indicated. Chromatin immunoprecipitation was performed using normal rabbit IgG or anti-TonEBP antibody. The *IFN-β* promoter region indicated was quantified using qPCR. Mean ± SEM, *n* = 3. (**D**) Cells were co-transfected for 24 h with various combinations of three plasmids expressing Flag-TonEBP, Flag-TonEBP Q1127A and SARS-CoV-2 NSP5, as indicated. Chromatin immunoprecipitation was performed as in (**C**). (**E**) Schematic of TonEBP NT (1–1127) and TonEBP sNT (1–548). (**F**) Cells were transfected for 24 h with the *IFN-β* promoter luciferase reporter plasmid and pRL-TK plus one of the combinations of plasmids expressing p65, TonEBP NT, and TonEBP sNT (1–548), as indicated. Luciferases were measured to assess the activity of *IFN-β* promoter. (**G**) Nucleotide sequence of a portion of the human *IFN-β* promoter for binding of IRF3 and NF-κB (p65). Sequences of three probes are shown below. PRDIII, PRDI, and PRDII regions are marked by yellow, green, and orange colors, respectively. (**H**,**I**) The probes shown above were biotinylated and individually immobilized on streptavidin sensor chips for surface plasmon resonance assay. Various concentrations of p65 (**H**) or TonEBP sNT (1–548) (**I**) recombinant proteins were injected over the sensor surface. Binding affinity was determined by plotting the maximum response unit versus the concentration of injected proteins using GraphPad Prism 9.0. The values of K_D_ are shown. (**J**) 100 nM each of Cy5.5-labeled *IFN-β* promoter probes were individually incubated without or with 500 nM TonEBP sNT (1–548) and resolved in an agarose gel. (**K**) Combinations of 0, 100, 200, or 500 nM TonEBP sNT (1–548) and p65 recombinant proteins (0 or 100 nM), as indicated, were each incubated for 25 min with 100 nM of Cy5.5-labeled IRF3/p65 probe and resolved in an agarose gel. The solid arrowhead denotes the complex of TonEBP sNT (1–548) with the probe and the open arrowhead denotes the complex of p65 with the probe. (**L**) A model for the IFN-β induction and its inhibition by TonEBP NT by replacing DNA bound p65. Data are shown as the mean ± SEM, *n* = 3; ns *p* > 0.05, and *** *p* < 0.001 using *t*-tests (**C**,**D**,**F**).

We set out to investigate how TonEBP NT suppress the *IFN-β* promoter. First, we asked whether TonEBP NT directly affected IRF3 or p65 by performing co-immunoprecipitation assay. While TonEBP NT did not interact with IRF3 (Figure 5A), it interacted with p65 (Figure 5B). To examine the TonEBP NT-p65 interaction on the *IFN-β* promoter in vivo, we performed chromatin immunoprecipitation assay. TonEBP binding to the *IFN-β* promoter increased 3 fold when TonEBP NT was expressed, and this binding was not affected by co-expression of MAVS (Figure 5C). Thus, it appears that TonEBP NT binds to the *IFN-β* promoter maximally irrespective of the activation of IRF3 and p65.

To examine the role of TonEBP cleavage by NSP5 on the TonEBP binding to the *IFN-β* promoter, we compared TonEBP vs. TonEBP Q1127A (Figure 5D). While TonEBP binding to the *IFN-β* promoter was comparable when TonEBP or TonEBP Q1127A was expressed, co-expression of NSP5 enhanced TonEBP binding significantly but had no effect on Q1127A. These data suggest that the production of TonEBP NT enhances TonEBP binding, i.e., TonEBP NT binds the promoter more efficiently than the full-length TonEBP.

We decided to perform biochemical analysis of TonEBP NT binding to the *IFN-β* promoter. We found that biochemical preparation of full-length TonEBP and TonEBP NT was difficult to obtain. On the other hand, a shorter N-terminal fragment of TonEBP including RHD but without TAD named TonEBP sNT (Figure 5E) can be produced. We speculate that the TAD is highly susceptible to proteolytic cleavage conferring biochemical instability likely because TAD is rich in Q (19.2%) and P (7.8%); removal of TAD altogether increases the stability as in TonEBP sNT. We found that TonEBP sNT suppressed the *IFN-β* promoter as well as TonEBP NT did (Figure 5F).

The core sequence of the *IFN-β* promoter for binding of IRF3 and p65 is shown in Figure 5G [17]. IRF3 binds both positive regulatory domain III (PRDIII) and PRDI, and p65 binds PRDII. To distinguish the binding of IRF3 and p65, we prepared three dsDNA probes: IRF3/p65 (full-length), IRF3 (PRDIII + PRDI for IRF3 binding), and p65 (PRDII for p65 binding). As expected, p65 probe and IRF3/p65 probe showed specific affinity to p65 with K_D_ of 35.2 nM and 11.5 nM, respectively (Figure 5H). Interestingly, TonEBP sNT displayed specific binding to both p65 probe and IRF3/p65 probe with K_D_ of 84.7 nM and 31.3 nM, respectively (Figure 5I). Thus, TonEBP sNT appeared to display specific affinity to PRDII like p65 with a reduced affinity. The affinity of TonEBP sNT to PRDII (probe IRF3/p65 and probe p65) was confirmed by electrophoretic mobility shift assay (EMSA) using an agarose gel (Figure 5J). To investigate the relationship between p65 and TonEBP sNT on the PRDII site, we performed EMSA using both proteins (Figure 5K). While both p65 and TonEBP sNT displayed clear binding to the IRF3/p65 probe, TonEBP sNT appeared to compete p65 away from PRDII forming a p65-TonEBP sNT complex as the concentration of TonEBP sNT increased. These data raise the possibility that displacement of p65 from PRDII by TonEBP sNT might be responsible for the suppression of the promoter by TonEBP sNT or TonEBP NT (see a model in Figure 5L).

## 4. Discussion

The data presented here demonstrate that NSP5s from HCoVs cleave TonEBP at amino acid position 1127 producing TonEBP NT. While TonEBP NT is incapable of enhancing the TonE-dependent transcription, it gains a new function to suppress the viral induction of IFN-β and, as a result, promote expansion of HCoV-OC43. A cleavage-resistant Q1127A mutant of TonEBP suppresses viral expansion in association with elevated IFN-β response (see more below). TonEBP adds to the repertoire of NSP5 targets to suppress the IFN-β induction by viral infection—RIG-I, eIF4G and NEMO [10,11,12].

How TonEBP NT gains the ability to suppress the IFN-β response is of great interest. Evidence for this new function is 2 fold. First is differential effects of TonEBP vs. a cleavage-resistant Q1127A mutants in viral expansion (Figure 3A–D) and IFN-β response (Figure 4G,H). The other is the dramatic effects of TonEBP NT in promoting viral expansion (Figure 3E–H) and suppressing the IFN-β response (Figure 4A–E). Biochemical analyses of TonEBP NT suggest that the cleavage confers the ability to suppress the transcriptional activity of NF-κB (p65) perhaps by competing away DNA binding (Figure 5K,L). This model is supported by elevated binding of TonEBP or TonEBP NT, but not by the Q1127A TonEBP, to the *IFN-β* promoter, which was measured using antibodies that detect both TonEBP NT and full-length TonEBP (Figure 5C,D).

TonEBP is also cleaved by CVB3 protease 2A at position 579 producing a protein similar to TonEBP sNT (see Figure 5E) [16]. This cleavage removes the entire TAD and inactivates the TonE-dependent transcriptional stimulation by TonEBP. As a result, inducible nitric oxide synthase expression is suppressed, leading to enhanced CVB3 replication and cardiac tissue damage because nitric oxide inhibits CVB3 replication by inactivating viral protease 2A and 3C [21]. Since the cardiac tissues are capable of IFN-β induction in response to CVB3 infection [22], we suspect that the N-terminal TonEBP fragment would also suppress the IFN-β induction like TonEBP sNT would (see Figure 5F) in the heart after CVB3 infection. It is likely that the cleavage of TonEBP promotes the viral expansion using multiple cellular pathways in the heart.

In macrophages and dendritic cells, infection by vesicular stomatitis virus or murine cytomegalovirus results in elevated TonEBP expression [17]. These viruses do not have genes encoding proteases. The elevated TonEBP, in turn, suppresses the IFN-β response by interfering with IRF3 binding. We also observed this effect when TonEBP was overexpressed (Figure 5G,H). Thus, TonEBP by itself (i.e., when it is not cleaved) is a proviral factor; myeloid deficiency of TonEBP is associated with elevated IFN-β response and reduced viral load in response to infection by lymphocytic choriomeningitis virus, which also does not encode a protease [17]. When infected by HCoV-OC43, TonEBP disappears in a time- and dose-dependent manner (Figure 1B,C). The disappearance of TonEBP should lead to elevated IFN-β response and suppression of viral expansion. But the cleavage of TonEBP produces TonEBP NT, which is another powerful anti-IFN-β factor with a new mechanism targeting NF-κB. The gain of function of TonEBP after the proteolytic cleavage compensates for the loss of full-length TonEBP to support viral expansion. Since this mechanism appears common to all the HCoVs tested, it is likely to be relevant to new variants and species of HCoVs that emerge in the future.

## 5. Conclusions

In this study, we uncovered a previously unrecognized strategy of coronaviruses to evade host immunity. Coronavirus NSP5 cleaves TonEBP to produce TonEBP NT, which gains the ability to compete p65 away from *IFN-β* promoter by directly binding to the PRD II domain on the *IFN-β* promoter. Displacement of p65 from *IFN-β* promoter by TonEBP NT decreases *IFN-β* expression to enhance coronavirus replication.

## Data Availability

All data associated with this study are present in this manuscript or Supplementary Materials.

## Supplementary Materials

The following supporting information can be downloaded at: https://www.mdpi.com/article/10.3390/cells13191614/s1, Figure S1: Sequence alignment of coronavirus NSP5. Figure S2: Site-specific cleavage of TonEBP at Q1127 by SARS-CoV-2 NSP5. Figure S3: TonEBP NT does not affect expression of proteins in the MDA5–IFN-β signaling pathway and phosphorylation of TBK1 and IRF3.
