# Supplementary material for: A Gain-of-Function Cleavage of TonEBP by Coronavirus NSP5 to Suppress IFN-β Expression
Source: Cells. 2024 Sep 26;13(19):1614. doi: 10.3390/cells13191614 (PMC11476177; doi:10.3390/cells13191614)
Supplement: Supplementary file 1 [file cells-13-01614-s001.zip › cells-3188079-supplementary.pdf]

## SUPPLEMENTAL INFORMATION

**This PDF file includes:**

Fig. S1. Sequence alignment of coronavirus NSP5.

Fig. S2. Site-specific cleavage of TonEBP at Q1127 by SARS-CoV-2 NSP5.

Fig. S3. TonEBP NT does not affect expression of proteins in the MDA5–IFN-β signaling pathway and phosphorylation of TBK1 and IRF3.

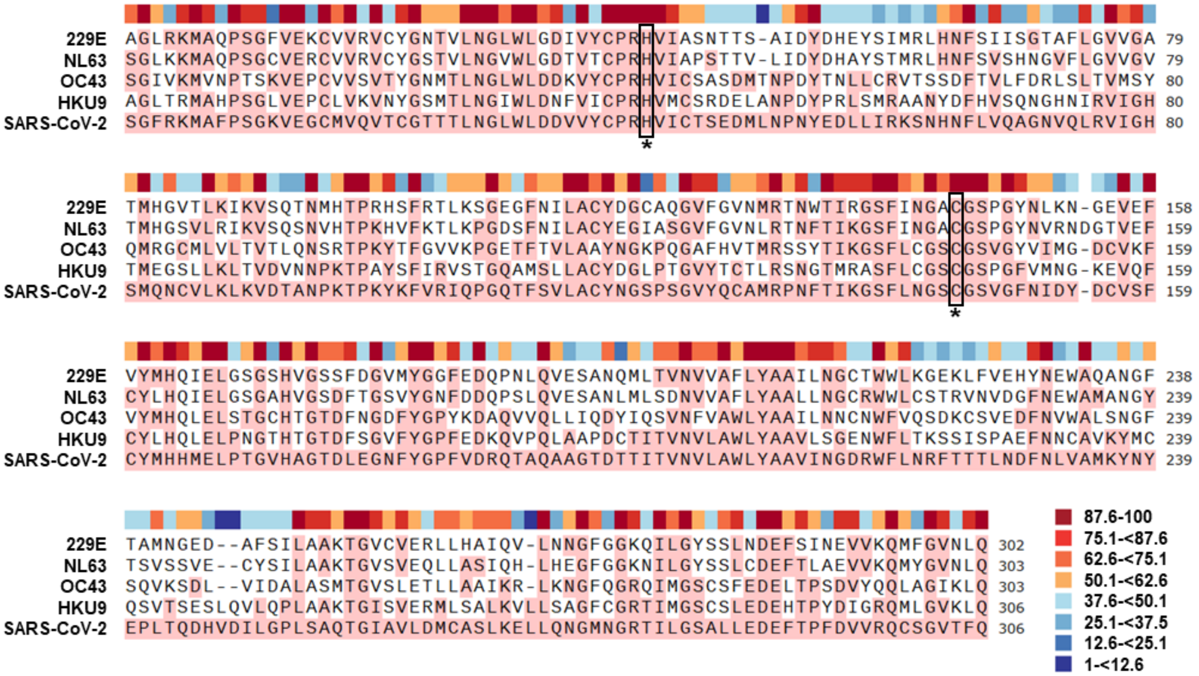


**Figure. S1** Sequence alignment of coronavirus NSP5. Sequences of human coronavirus 229E, NL63, OC43, bat coronavirus HKU9, and SARS-CoV-2 NSP5 were aligned. The catalytic dyad residues (His/Cys) are boxed. The amino acids identified to SARS-CoV-2 is marked by pink and conserved amino acids are shown with color score range.

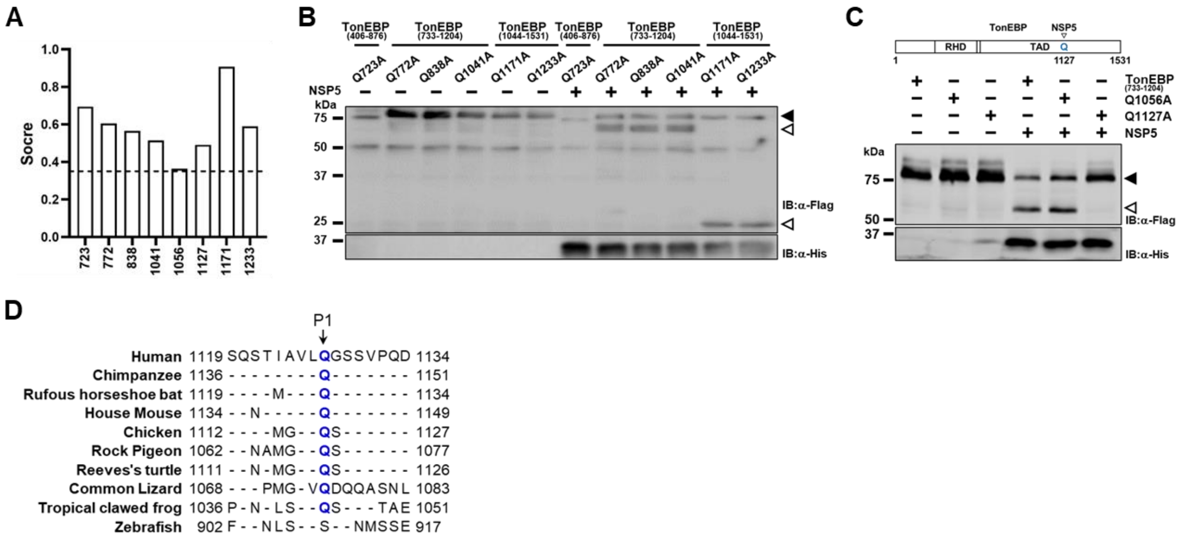


**Figure. S2** Site-specific cleavage of TonEBP at Q1127 by SARS-CoV-2 NSP5. (**A**) NetCorona 1.0 prediction of the potential TonEBP cleavage sites used by SARS-CoV-2 NSP5. The dotted line indicates the threshold (0.35). The predicted sites of TonEBP have been arranged in the order of the amino acid sequence number of TonEBP. (**B**) HEK293T cells were transfected with plasmids expressing Flag-tagged truncated TonEBP at positions indicated which were mutated at the indicated putative cleavage site, and His-tagged SARS-CoV-2 NSP5. Solid arrowhead denotes truncated TonEBP, whereas open arrowhead denotes cleavage product. (**C**) Schematic of TonEBP showing the location of the NSP5 cleavage site (top). Cells were transfected with various combinations of expression plasmids for Flag-TonEBP (733–1204) wild type, Q1026A mutant, and Q1127A, and His-tagged SARS-CoV-2 NSP5. (**D**) Amino acid sequence alignment of the region of TonEBP targeted by NSP5 from various species. The putative cleavage sites (Q) are in blue and labeled as P1.

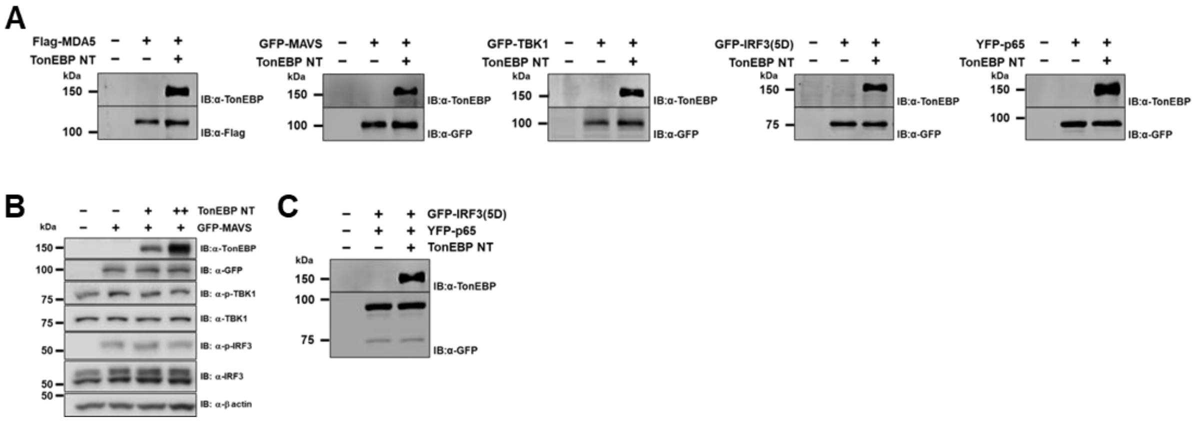


**Figure. S3** TonEBP NT does not affect expression of proteins in the MDA5–IFN-β signaling pathway and phosphorylation of TBK1 and IRF3. (**A**) HEK293T cells were transfected with either an empty vector or a plasmid expressing Flag-TonEBP NT, along with plasmids expressing Flag-MDA5, GFP-MAVS, GFP-TBK1, GFP-IRF3-5D, or YFP-p65. (**B**) Cells were transfected for 24 h with a plasmid expressing GFP-MAVS along with various amounts of plasmids expressing Flag-TonEBP NT. Phosphorylated TBK1 and IRF3 were immunoblotted. (**C**) Cells were transfected with various combinations of plasmids expressing TonEBP NT, GFP-IRF3-5D and YFP-p65, as indicated.
